# The effectiveness of an m-Health intervention on the sexual and reproductive health of in-school adolescents: a cluster randomized controlled trial in Nigeria

**DOI:** 10.1186/s12978-023-01735-4

**Published:** 2024-01-13

**Authors:** Oluwatosin Wuraola Akande, Moise Muzigaba, Ehimario Uche Igumbor, Kelly Elimian, Oladimeji Akeem Bolarinwa, Omotosho Ibraheem Musa, Tanimola Makanjuola Akande

**Affiliations:** 1 Department of Epidemiology and Community Health, University of Ilorin Teaching Hospital, Ilorin, Kwara State Nigeria; 2 Independent Scientist, Geneva, Switzerland; 3 Centre for Infectious Disease Research, Nigerian Institute of Medical Research, Lagos, Nigeria; 4 School of Health Systems and Public Health, University of Pretoria, Pretoria, South Africa; 5 Department of Microbiology, Faculty of Life Sciences, University of Benin, Benin City, Nigeria; 6 Department of Global Public Health, Karolinska Institutet, Stockholm, Sweden

**Keywords:** mHealth, Adolescents, Sexual and reproductive health, Cluster randomized controlled trial

## Abstract

**Background:**

The implementation of the country-wide comprehensive sexuality education (CSE) curriculum among in-school adolescents remains abysmally low and mHealth-based interventions are promising. We assessed the effect of a mHealth-based CSE on the sexual and reproductive health (SRH) knowledge, attitude and behaviour of in-school adolescents in Ilorin, northcentral Nigeria.

**Methods:**

Using schools as clusters, 1280 in-school adolescents were randomised into intervention and control groups. Data was collected at baseline (T_0_), immediately after the intervention (T_1_) and 3 months afterwards (T_2_) on SRH knowledge, attitude and practice of risky sexual behaviour (RSB). Data analysis included test of associations using Chi-square, independent t-test and repeated measures ANOVA. Predictors were identified using binary logistic regression.

**Results:**

In the intervention group, there was a statistically significant main effect on mean knowledge score (F = 2117.252, p =  < 0.001) and mean attitude score (F = 148.493, p =  < 0.001) from T_0_ to T_2_ compared to the control group which showed no statistically significant main effects in knowledge (p = 0.073), attitude (p = 0.142) and RSB (p = 0.142). Though the mean RSB score declined from T_0_ to T_2_, this effect was not statistically significant (F = 0.558, p = 0.572). Post-intervention, being female was a positive predictor of good SRH knowledge; being male was a positive predictor of RSB while being in a higher-class level was a negative predictor of RSB.

**Conclusion:**

The mHealth-based CSE was effective in improving SRH knowledge and attitude among in-school adolescents. This strategy should be strengthened to bridge the SRH knowledge and attitude gap among in-school adolescents.

*Trial registration* Retrospectively registered on the Pan African Clinical Trial Registry (pactr.samrc.ac.za) on 19 October 2023. Identification number: PACTR202310485136014

**Supplementary Information:**

The online version contains supplementary material available at 10.1186/s12978-023-01735-4.

## Plain Language Summary

In Nigeria, the implementation of a nationwide sex education programme for adolescents going to schools is below expectation but using mobile health (mHealth) interventions could help. In this study, we looked at how a mHealth-based sex education programme affected the sexual and reproductive health (SRH) knowledge, attitude, and behaviour of in-school adolescents in Ilorin, Nigeria. We divided 1280 students into two groups, one received the mHealth-based intervention and the other did not receive it. We collected data before the intervention, right after it, and 3 months later to see any changes in SRH knowledge, attitudes, and risky sexual behaviours. We used various statistical tests to analyze the data and find patterns. The results showed that the group that received the mHealth intervention had significant improvements in their knowledge and attitudes about SRH from the start of the study to 3 months after the intervention. However, the control group, which didn't get the intervention, didn't show these improvements significantly. While the risky sexual behaviour score decreased slightly in the intervention group, this change was not significant. After the intervention, we found that being female was associated with better SRH knowledge, while being male was linked to more risky sexual behaviours. Also, being in a higher class level was associated with low risky behaviour. In conclusion, using mHealth for sex education helped improve the SRH knowledge and attitudes of students. This approach could be scaled to fill the gap in SRH knowledge and attitudes among adolescents in schools.

**Supplementary Information:**

The online version contains supplementary material available at 10.1186/s12978-023-01735-4.

## Background

Globally, adolescents aged 10 to 19 years make up about 16% of the population [1]. This age group makes up a higher proportion of sub-Saharan Africa and Nigeria, accounting for 23% and 22.3% respectively [1, 2]. Adolescence is a period of rapid human development which includes physical, neurodevelopmental, psychological, and social changes with implications for their peculiar health needs. The majority of the serious health challenges in adulthood have roots in the period of adolescence, and about 70% of premature deaths among adults are mostly related to behaviours initiated at adolescence [1].

Sexual and Reproductive Health (SRH) issues, including sexually transmitted infections and unintended pregnancies, account for a significant proportion of disease burden among adolescents [3]. A 12-year review of Nigerian adolescents sexual practices and behaviours found that they engage in risky sexual behaviours consisting of early sexual debut, unsafe sexual practices, and concurrent multiple sexual partners [4].

There is growing evidence to show that SRH of adolescents can be improved through Comprehensive sexuality education (CSE) [5]. The CSE curriculum may also be known as "life skills," "family life," or "HIV education” or "holistic sexuality education" implying the difference in the emphasis of the curricula [6]. The policy of the Nigerian government at the national level identifies the pressing SRH needs of adolescents and has acted on its policy commitments by implementing a near-nationwide CSE. Family Life and HIV Education (FLHE) is the form of CSE being implemented by the government into school curricula at the basic and secondary school levels in Nigeria, in addition to teacher's training institutions [7]; its main aim is to prevent HIV/AIDS through awareness and education.

Given the limitations associated with the delivery of FLHE in Nigeria which is mainly via didactic physical lectures, and consequently, low nation-wide implementation and uptake, there is a need for more innovative and effective strategies to reach these adolescents [7, 8]. mHealth is one of such innovation with the potential of wider acceptability by the adolescent population. mhealth is the use of "emerging mobile communications and network technologies for healthcare" and it has gained prominence in recent years [9]. Globally, mobile phone subscriptions have been exponentially increasing, especially in developing countries where mobile subscriptions increased from 1.2 billion in 2005 to over 5.5 billion in 2015 [10]. A study done among 726 females between the ages of 12 and 30 years in six states in Nigeria showed that about 98.6% of them had access to a mobile phone [11]. Another study conducted among 249 in-school teenagers in Enugu State in southeast Nigeria found that about 69% of them had access to the internet via their phones, laptops and tablets [12].

Adolescents use the internet for their health-related needs, and the proportion who use this service is projected to increase in the next few years [13, 14]. Many adolescents cannot discuss SRH issues with their parents due to poor communication and cultural norms on sexuality issues, and they would rather rely on information from the internet or their peers who may have incorrect or inadequate information [15].

Within the context of the current gaps in the delivery of the FLHE in Nigeria and the revolutionization of information access through mHealth in the country, we developed and implemented a mHealth-based CSE curriculum over 12 weeks and assessed its effect on the SRH, attitude, and sexual behaviour of in-school adolescents in Ilorin, Nigeria.

## Methods

### Trial design

A two-arm Cluster Randomized Controlled Trial (cRCT) of 8 schools (clusters) with equal allocation was conducted. This number meets the minimum number of clusters required for cRCTs [16]. Individual students served as participants and outcome measures were at the individual participant level. SRH knowledge, attitude and sexual behaviour were assessed at baseline (T_0_), immediately after the 12-week intervention (T_1_), and 3 months after the intervention (T_2_).

### Study setting

The study was conducted between 10th of February 2020 and 28th of August 2020 in secondary schools located in Ilorin, Kwara State, Nigeria. Ilorin is the capital city of Kwara State and has a youth literacy rate of 76.9% and total gross school enrolment ratio of 50.13% (52.57% for males and 47.64% for females) [17]. One of the focus areas of the National School Health Programme by the Federal Ministry of Education is the provision of skill-based education and FLHE is part of the skill-based curriculum [18].

#### Eligibility criteria for schools

To be eligible to participate, schools had to be registered with the Kwara State Ministry of Education. Secondary school commences after 5–6 years of primary (elementary) school, and the system is divided into the junior secondary school (year 1–3) and senior secondary school (year 3–6).

#### Eligibility criteria for students

In-school adolescents (aged 10–19 years) in senior secondary school and who had access to the internet at least once a week throughout the study duration were eligible to participate. The students either owned these devices or had access to these devices through their parents/guardians.

Students who had cognitive or visual impairments were excluded from the trial.

### Group assignment and masking

**Fig. 1:**
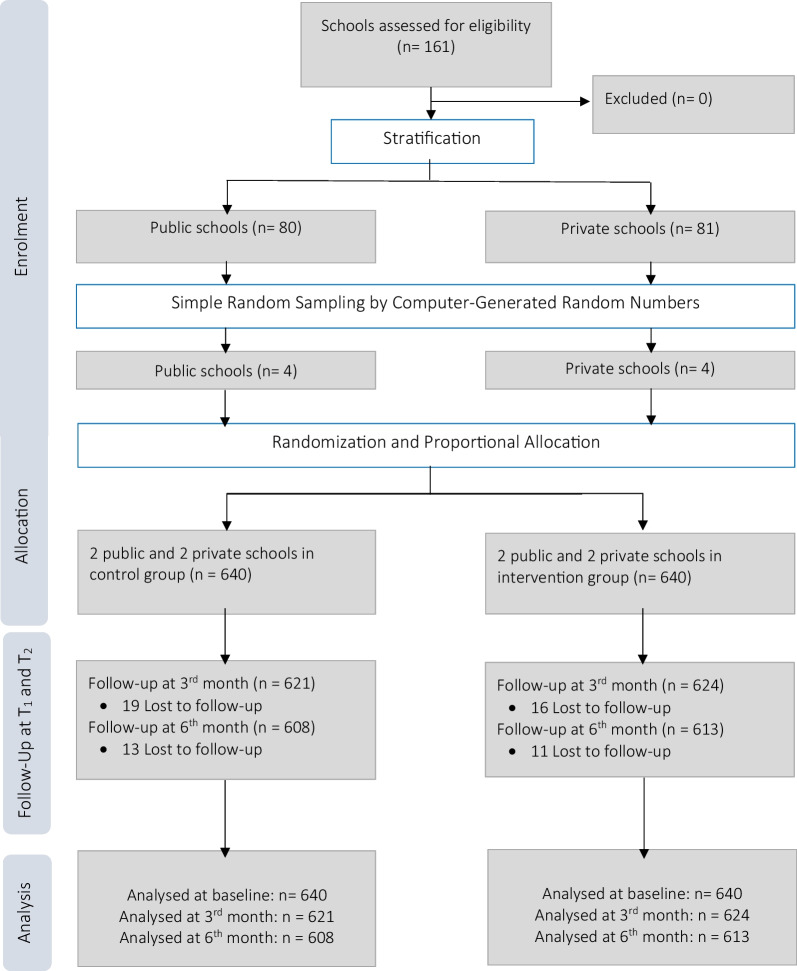
Consort flowchart for the trial

Eligible schools (n = 161) were stratified into public (n = 80) and private schools (n = 81). Eight schools (4 public schools and 4 private schools) were selected using simple random sampling by computer-generated random numbers (Fig. 1). Schools were then assigned to a cluster design to avoid contamination bias following consent from the school principals. Researchers were not blinded to the assignment, but students were not informed of their school’s allocation. To reduce contamination bias in this study, schools formed cluster units for allocation into study groups and we ensured they were at least 40 m apart.

### Sample size and sampling strategy

The target sample size for this study was 1280 participants (640 per group) from 8 schools. The superiority trial formula for continuous variables which is used to verify that a new intervention is more effective than the usual intervention from a statistical/clinical point of view was used to calculate the sample size [19]. The statistical power was set at 0.80, alpha at 0.05 and attrition rate of 10% among participants. Mean scores in the control and intervention group were set at 16.61 and 17.47 using a previous study [20]. A design effect of 2 was calculated assuming an intraclass correlation of 0.05 and number of individuals per cluster of 21 to allow for possible clustering effect.

### Recruitment and consent/assent

Based on the student enrolment profile of each school (Additional file 1), proportional allocation was used to allocate sample size to each of the selected schools based on their population in the first stage. In the second stage, disproportionate stratified sampling for between-strata analysis which is used to maximize sample size of each stratum using equal allocation for comparative analysis was employed [23, 24]. In the third stage, using the nominal roll which contains the list of students in each class, participants in each class were selected using a systematic sampling technique.

A letter of introduction was obtained from the Department of Epidemiology and Community Health, University of Ilorin Teaching Hospital and the Kwara State Ministry of Education to the principals of the schools selected. Prior to the commencement of the study, multiple advocacy visits were paid to the principals, head teachers and others in authority in the selected schools. The visits involved discussions about the study objectives and the link to the government-approved FLHE curriculum, data collection methodology and timeframe, parental/guardian consent forms with the study information leaflet, study questionnaire, etc.

Following adequate briefing and the approval to conduct the study in their school, the principal/head teacher or designated officers introduced the study and the research team to the students. Class-to-class interactive sessions about the study were conducted. Simplified study information leaflets were also distributed to the students and there were opportunities for them to ask questions during the sessions. The selected respondents were given forms which included the study information leaflet to obtain signed written consent from one of their parents or a guardian at least 2 weeks to the commencement of data collection.

In Nigeria, a minor is defined as one who is below the age of 18 years. In this study, those who were 18 years and above gave written consent to participate in the study by themselves. For those less than 18 years, only those who submitted a consent form signed by a parent or guardian and gave verbal assent to participate in the study (obtained on the first day of data collection and witnessed by the research team) were recruited into the study.

### Intervention

Schools allocated to the intervention group were given access to the mHealth-based CSE, which contained 12 modules via accessible online (link: http://flhe.noubug.com) over a 12-week period (24th February 2020 to 23rd May 2020). The 12-module CSE was an adoption of the approved FHLE curriculum for secondary schools in Nigeria that covered six themes: human development, personal skills, sexual health relationships, sexual behaviour, and society and culture [22]. Topics across these six themes were covered over the 12-week period (Additional file 2). Each participant was given a username (not linked to any personal identifier) and password (which each user could change) to access the CSE curriculum online. Participants could ask questions anonymously via the website, and responses were given within 24 h. During this period, a total number of 51 questions were asked by 47 respondents. Majority of the questions, 38 (80.9%) were related to the course contents while 9 (19.1%) were questions requesting for technical support in navigating the site. Participants in both public and private schools were not provided with free data to browse the internet for this study but used their existing internet data sources prior to the study. This was done to assess the sustainability of in-school adolescents utilising mHealth-based interventions without the availability of incentives such as the provision of free data for browsing.

### Control

The control was a 12-week school-as-usual condition. Participants in the control group were not exposed to the mHealth-based intervention. Instead, they were to continue with the usual classroom-based CSE according to the existing school curriculum during the intervention period. However, due to the coronavirus disease 2019 (COVID-19) pandemic, all schools in study area were shut during this period, and this disrupted the regular educational routine of these students.

### Study instrument

We used a questionnaire adapted from the World Health Organisation’s questionnaire for collecting data on SRH behaviours [23]. The questions were modified based on the modules covered in the intervention. Section 1 addressed the respondents’ sociodemographic characteristics; Sections 2, 3, 4 and 5 assessed the respondents’ SRH knowledge, attitude and sexual behaviour respectively. The questionnaire was pre-tested among students of two senior secondary schools (one public, one private) other than the selected schools (n = 128). The schools chosen for the pre-test were at least 10km from the study and control schools.

All study tools were tested for accuracy and content validity through the consultation of relevant literature on SRH education for adolescents. They were also reviewed by academic experts, including eight Consultant Public Health Physicians with expertise in SRH, for its content and structure validity. The coefficient of internal reliability analysis of the tool was 0.757, which is fairly high [24]. Pre-testing helped determine its level of difficulty, complexity, logical sequence, spot inconsistencies, and standardise questionnaire administration language and style.

### Data collection

Data was collected by Six Research Assistants (RAs) who were trained on the content and administration of the research instrument. Three RAs were Medical Officers in Ilorin, and the other three were adolescents between the ages of 18 and 19 years. Baseline assessment (T_0_) was done using paper-based pre-tested interviewer-led, self-administered questionnaires in classrooms under the guidance of the Lead Researcher (OWA) and the RAs (10th–22nd of February 2020).

Attitudinal and behavioural change among adolescents takes substantial time [25]. Thus, to give ample time to measure the effect of the intervention among the respondents, all students in both groups were followed up to assess SRH knowledge, attitude towards SRH and sexual behaviour immediately after the intervention (T_1_) and 3 months after the end of the intervention (T_2_). Post assessment data at T_1_ (24th May 2020–5th June 2020) and T_2_ (17th August 2020–28th August 2020) were collected using the pre-tested interviewer-led, self-administered questionnaire administered at baseline.

Due to the COVID-19 pandemic, schools in the study area were temporarily shut down on 23rd March, 2023, 4 weeks into the study. Following the announcement and before the schools were closed, a visit was made to all the schools to inform the respondents about the use of an online Google form for the collection of the post-intervention data. Thus, T_1_ and T_2_ data were collected online from both the control and intervention groups using a Google form. To maximize response rate, text messages which included the link to the questionnaire were sent to all respondents. Furthermore, class representatives who were selected in each class of all the schools were urged to remind and encourage their peers to fill the online questionnaire using their existing WhatsApp platforms.

Following the final assessment at T_2_, all respondents (including those in the control group) were given access to the CSE via the website for 4 months.

### Outcome measures

The primary outcome was participants mean scores in SRH knowledge, SRH attitude and RSB of participants, measured at baseline, T_1_ and T_2_.

#### Computation of composite scores

Section 2 of the study questionnaire contained 65 multiple choice questions that covered the knowledge assessment of SRH. These questions covered knowledge on puberty and pubertal changes, reproductive health, sexually transmitted infections (STIs) including human immunodeficiency virus (HIV), acquired immunodeficiency syndrome (AIDS) and modern contraceptives. Based on the core questionnaire measurement and reference to similar research that adapted the same instrument, a score of 1 was assigned to every correct answer, while a score of 0 was assigned to every incorrect answer [20]. Thus, the maximum score for knowledge was 65 points, and the minimum score was 0 point. The scores were summed up and converted to 100%. Mean scores were calculated for both groups. Also, the individual scores were categorised into 3: good (> 66%), fair (34.0–65.9%) and poor (< 34%). This is as categorised in a previous study that assessed the SRH knowledge of adolescents in Ibadan, Nigeria [26].

Section 3 of the questionnaire focused on the attitudinal assessment of SRH. It consisted of a list of 13 statements describing attitudinal disposition (such as their perception towards premarital sex, contraceptive use, and sex education) which were answered on a 5-point Likert scale (1—agree a lot, 2—agree, 3—indifferent, 4—disagree, 5—disagree a lot). Each item was rated 1 to 5 with total scores ranging from 13 to 65. Questions 44, 45 and 46 were reverse scored. The items were summed up and converted to 100%. Mean scores were obtained in both groups. Individual scores were also categorised into 2: positive (≥ 50%) and negative (< 50%). This is as categorised in a previous study that assessed the attitude of adolescents towards SRH [27].

Section 4 of the questionnaire consisted of 15 questions regarding sexual behaviour. The first item asked respondents if they were sexually active. The prevalence of risky sexual behaviour was defined as reporting one or more of the following: multiple sexual partners, exchange of material gift or money for sex, inconsistent/incorrect/non-use use of condoms at least once during sexual intercourse, getting infected by an STI, and sexual debut before the age of 18 years [28]. An affirmative answer to any of the questions was scored one. Thus, the total scores for risky sexual behaviour ranged from 1 to 5. Those who did not report any of the listed behaviour were categorised as practising protective sexual behaviour, while those who affirmed to practising any of the listed behaviour were categorised as practising risky sexual behaviour. The prevalence of risky sexual behaviour was calculated, and mean scores were also calculated in both groups.

Among respondents in the intervention group, uptake of CSE was scored using the number of modules completed at P1 and P2. Completion of each module was given a score of 1. Number of completed modules were summed up and converted to 100%. Mean scores were also calculated among respondents in private and public schools.

In addition, we identified factors influencing the primary outcomes (SRH knowledge, attitude and sexual behaviour) using multivariate analysis using binary logistic regression.

### Statistical analysis

Statistical analyses were performed using StataCorp. 2019. Stata Statistical Software: Release 16. College Station, TX: StataCorp LLC. Data visualisations were created using R-Studio Version 1.3.1073.

All continuous data were first tested for normality using the Shapiro–Wilk and Shapiro-Francia tests. All continuous variables including scores for dependent variables were normally distributed and thus mean and standard deviation were used as summary statistics. Respondents’ baseline socio-demographic characteristics measured as categorical variables were summarized using frequencies and percentages and presented in tabular form. The between-group differences in the distribution of continuous data were visually inspected using box plots and statistically compared using the independent samples t-test. Pearson’s Chi Square and Fisher’s exact tests were used to assess whether there are statistically significant relationships between categorical predictor variables and categorical outcome variables.

The predictor variables which yielded a p-value less than 0.25 during bivariate analysis were used for multivariate binary logistic regression analysis for the identification of factors influencing SRH knowledge, attitude, and sexual behaviour. In the multivariate model, factors associated with dependent variables were evaluated using adjusted Odds Ratios (AORs) and 95% Confidence Intervals (CI). For the AOR estimator, the Hosmer–Lemeshow test was used to determine the model’s goodness of fit with the likelihood ratio test as a primary measure of model fit. The relative importance of individual predictors in the model were assessed using the t-statistic for each model parameter. The main analysis was intention-to-treat based on the randomisation of clusters. Repeated measures ANOVA was used in assessing the effectiveness of the study intervention. Throughout the analysis, a p-value < 0.05 was considered statistically significant.

### Patient and public involvement statement

This trial did not involve patients but rather in-school adolescents. The intervention developed upon an already existing programme targeting in-school adolescents. The choice of an mHealth intervention is premised on the interest and high levels of update of mobile technology by adolescents. In developing the intervention, a pilot study was conducted which enabled incorporation of the inputs of adolescents as users of the intervention. Furthermore, adolescents were included among the data collectors.

## Results

### Characteristics of the participants

More than half of the respondents in both groups were in the age range 15–17 years (Table 1). The proportion of males and females in both groups were almost equal. Public school enrolment accounted for 480 (75.0%) and 459 (71.7%) in the control and intervention groups respectively. More than two thirds of the respondents had been exposed to sexuality education at home, accounting for 475 (74.2%) and 468 (73.1%) in the control and intervention groups respectively. For all the aforementioned variables, there were no statistically significant differences between the two study groups thereby confirming group equivalence due to effective randomization.

**Table 1:** Characteristics of respondents

Variable	Control (%)	Intervention (%)	Total (%)	χ^2^/t	p value
	n = 640	n = 640	n = 1280		
*Respondents’ characteristics*					
Age groups (years)				2.002	0.368
12–14	204 (31.9)	208 (32.5)	412 (32.2)		
15–17	366 (57.2)	377 (58.9)	743 (58.0)		
> 17	70 (10.9)	55 (8.6)	125 (9.8)		
Mean age ± SD	15.25 ± 1.69	15.21 ± 1.65	15.23 ± 1.67	0.452	0.651
Gender				31.3	0.576
Female	324 (50.6)	314 (49.1)	638 (49.8)		
Male	316 (49.4)	326 (50.9)	642 (50.2)		
School type				1.763	0.184
Public	480 (75.0)	459 (71.7)	939 (73.4)		
Private	160 (25.0)	181 (28.3)	341 (26.6)		
Class				0.0005	0.998
SS1	214 (33.4)	215 (33.6)	429 (33.5)		
SS2	214 (33.4)	214 33.4)	428 (33.4)		
SS3	212 (33.1)	211 (33.0)	423 (33.1)		
Subject combination				5.763	0.056
Science	238 (37.2)	236 (36.9)	474 (37.1)		
Art	201 (31.4)	236 (36.9)	437 (34.1)		
Commercial	201 (31.4)	168 (26.2)	369 (28.8)		
Tribe				7.149	0.067
Yoruba	507 (79.2)	531 (83.0)	1038 (81.0)		
Hausa	27 (4.3)	33 (5.2)	60 (4.7)		
Igbo	31 (4.8)	17 (2.7)	48 (3.8)		
Others	75 (11.7)	59 (9.2)	134 (10.5)		
Marital status				5.321	0.070
Single	628 (98.1)	614 (95.6)	1242 (97.0)		
Married	4 (0.6)	9 (1.4)	13 (1.0)		
Others	8 (1.3)	17 (2.7)	25 (2.0)		
Religion				3.516	0.061
Christianity	240 (37.5)	208 (32.5)	448 (35.0)		
Islam	400 (62.5)	432 (67.5)	832 (65.0)		
Who respondents lived with				6.341	0.092
Parents	532 (83.1)	563 (88.0)	1095 (88.5)		
Other relatives	87 (13.6)	63 (9.8)	150 (11.7)		
Alone	14 (2.2)	8 (1.3)	22 (1.7)		
Others	7 (1.1)	6 (0.9)	13 (1.0)		
*Respondents’ family characteristics*					
Type of family				3.529	0.060
Polygamous	181 (28.3)	212 (33.1)	393 (30.7)		
Monogamous	459 (71.7)	428 (66.9)	887 (69.3)		
Respondents’ marital status				5.321	0.070
Single	628 (98.1)	614 (95.6)	1242 (97.0)		
Married	4 (0.6)	9 (1.4)	13 (1.0)		
Others	8 (1.3)	17 (2.7)	25 (2.0)		
Parents marital status				6.679	0.083
Married	542 (84.7)	569 (88.9)	1111 (86.8)		
Separated	65 (10.2)	40 (6.3)	105 (8.2)		
Widowed	22 (3.4)	21 (3.3)	43 (3.4)		
Divorced	11 (1.7)	10 (1.6)	21 (1.6)		
Father’s employment status				4.138	0.126
Unemployed	28 (4.4)	44 (6.9)	72 (5.6)		
Self employed	374 (58.4)	354 (55.3)	728 (56.9)		
Civil servant	238 (37.2)	242 (37.8)	480 (37.5)		
Mother’s employment status				4.850	0.088
Unemployed	44 (6.9)	46 (7.2)	90 (7.0)		
Self employed	333 (52.0)	369 (57.7)	702 (54.8)		
Civil servant	263 (41.1)	225 (35.1)	488 (38.1)		
Number of siblings				7.804	0.050
0	16 (2.5)	4 (0.6)	20 (1.6)		
1–3	255 (39.8)	252 (39.4)	507 (39.6)		
4–6	310 (48.4)	317 (49.5)	627 (49.0)		
7–18	59 (9.2)	67 (10.5)	126 (9.8)		
Sexuality education at home	475 (74.2)	468 (73.1)	337 (26.3)	0.197	0.657
Sexuality education in school	538 (84.1)	524 (81.1)	1062 (83.0)	1.084	0.298

χ^2^—Pearson’s Chi square test; t—independent t test; level of significance—p value < 0.05

### Baseline SRH knowledge, attitude, and RSB

Most respondents (63.9%) had a fair knowledge of SRH, accounting for 401 (62.7%) and 417 (65.2%) in the control and intervention groups respectively (Table 2). The mean knowledge score was 62.67 (SD = 9.90) in the control group and 61.97 (SD = 10.35) in the intervention group (p value = 0.218). Furthermore, most of the respondents had a positive attitude towards SRH, accounting for 475 (74.2%) and 483 (75.5%) in the control and intervention groups respectively (p value = 0.607). The mean attitude score was 64.54 (SD = 20.48) in the control group and 75.46 (SD = 18.32) in the intervention group (p value = 0.063). The prevalence of RSB was found to be 9.7% in the control group and 9.2% in the intervention group at baseline. Among those who were sexually active, almost all of them practised risky sexual behaviour, accounting for 86.1% and 93.5% in the control and intervention groups respectively. Regarding the mean score of RSB, the scores at baseline in the control and intervention groups were 4.69 (SD = 15.56) and 4.66 (SD = 14.42) respectively. There was no statistically significant difference in SRH knowledge, attitude and risky sexual behaviour between both groups.

**Table 2:** Baseline profiles for SRH knowledge, attitude, and risky sexual behaviour

Variable	Control (%)	Intervention (%)	Total (%)	χ^2^/t	*p* value
	n = 640	n = 640	n = 1280		
SRH knowledge at baseline					
				1.527	0.466
Poor knowledge	3 (0.4)	5 (0.8)	8 (0.6)		
Fair knowledge	401 (62.7)	417 (65.2)	818 (63.9)		
Good knowledge	236 (36.9)	218 (34.0)	454 (35.5)		
Mean score (SD)	62.67 (9.90)	61.97 (10.35)	62.32 (10.13)	1.234	0.218
SRH attitude at baseline					
				0.266	0.606
Negative attitude	165 (25.8)	157 (24.5)	322 (25.2)		
Positive attitude	475 (74.2)	483 (75.5)	958 (74.8)		
Mean score (SD)	64.54 (20.48)	75.46 (18.32)	70.00 (19.40)	1.859	0.063
RSB at baseline					
Practice of RSB	62 (9.7)	59 (9.2)	121 (9.5)	0.082	0.774
Mean score (SD)	4.69 (15.56)	4.66 (14.42)	4.68 (14.99)	− 0.37	0.788

χ^2^—Pearson’s Chi square test; t—independent t test; level of statistical significance—p value < 0.05

### Intervention effect

**Fig. 2:**
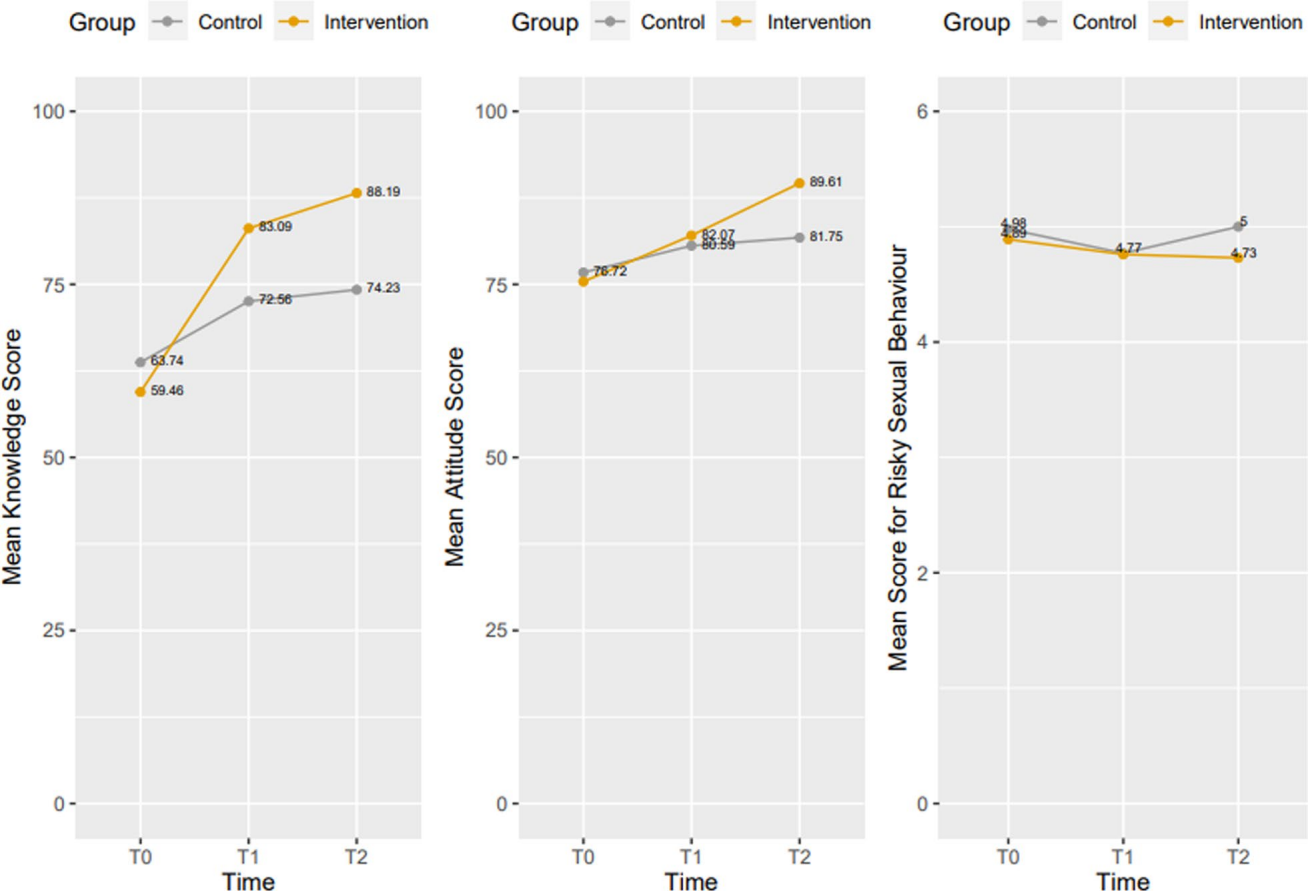
Effect of the mHealth-based Intervention on knowledge, attitude and sexual behaviour in the control and intervention groups: repeated Measures graphical illustration

In the intervention group, uptake rates (completion of at least 75% of the mHealth-based curriculum and 100% completion of the questionnaire) at T_1_ and T_2_ were 94.9% and 97.5% respectively. Table 3 presents the results from the Repeated Measures ANOVA to assess the effect of the intervention on knowledge, attitude and sexual behaviour in the study groups. Figure 2 provides a graphic illustration of these results.

**Table 3:** Effect of the mHealth-based Intervention on knowledge, attitude and sexual behaviour in the control and intervention groups: repeated measures ANOVA estimates

	Control Group				Intervention group			
	Mean ± SD	F Ratio	*p* value	ηp^2^	Mean ± SD	F Ratio	*p* value	ηp^2^
SRH knowledge		21.459	0.073	0.014		2117.252	**< 0.001**	0.776
T_0_	63.74 ± 10.10				59.46 ± 13.99			
T_1_	72.56 ± 13.76				83.09 ± 12.98			
T_2_	74.23 ± 14.03				88.19 ± 9.45			
SRH attitude		12.203	0.142	0.012		148.493	**< 0.001**	0.195
T_0_	76.72 ± 17.15				75.46 ± 15.87			
T_1_	80.59 ± 15.23				82.07 ± 20.46			
T_2_	81.75 ± 14.80				89.61 ± 10.19			
Risky sexual behaviour		5.769	0.572	0.009		0.558	0.572	**0.001**
T_0_	4.98 ± 15.50				4.89 ± 15.87			
T_1_	4.77 ± 14.87				4.76 ± 15.50			
T_2_	5.01 ± 12.78				4.73 ± 15.48			

SD: Standard deviation; ηp^2^: Partial eta square; level of statistical significance—p < 0.05 (bold)

The analysis shows that in the control group there were no statistically significant changes in the mean SRH knowledge score, the mean SRH attitude score, and the mean RSB score (p = 0.073, 0.142 and 0.572 respectively) from T_0_ to T_2_. However, in the intervention group, there was a statistically significant main effect of the mHealth-based intervention on the mean knowledge score [F (1.431, 875.761) = 2117.252, ρ =  < 0.001, ηp2 = 0.776). Bonferroni post hoc tests showed that the respondents had significantly higher mean knowledge score at T_0_, compared to T_1_ (59.45 ± 13.99 versus 83.09 ± 12.98, respectively; ρ =  < 0.001). At T_2_, the mean knowledge score increased to 88.19 (SD = 9.45), which was significantly higher than the mean at T_0_ (ρ =  < 0.001) and T_1_ (ρ =  < 0.001). Similarly, the intervention has a statistically significant effect on the mean attitude score [ F (1.485, 908.885) = 148.493, ρ =  < 0.001, ηp2 = 0.195) and the Bonferroni post hoc tests showed that the respondents had significantly higher mean attitude score at T_0_, compared to T_1_ (75.46 ± 18.32 versus 82.07 ± 20.46, respectively; ρ =  < 0.001). At T_2_, mean attitude score increased to 89.61 ± 10.19, which was significantly higher than the mean at T_0_ (ρ =  < 0.001) and T_1_ (ρ =  < 0.001). Nevertheless, even though the mean RSB score declined from T_0_ to T_1_ in the intervention group (4.89 ± 15.87 versus 4.76 ± 15.50, respectively) and again at T_2_, (4.73 ± 15.48), these improvements were not statistically significant [F (2, 1224) = 0.558, ρ = 0.572, ηp2 = 0.001)].

### Predictive analysis

As shown in Table 4, gender (p = 0.012) and type of school (p = 0.001) were significantly associated with knowledge. Age range was also found to be significantly associated with attitude (p = 0.003). Age, gender, class, and father’s employment type were statistically associated with RSB (p < 0.001; p < 0.001; p = 0.004; and p < 0.001 respectively).

**Table 4:** Univariate analysis of sociodemographic factors associated with respondents’ SRH knowledge, attitude and SRB, post Intervention

Variable	Knowledge		Attitude		Behaviour	
	Fair (%)	Good (%)	Negative (%)	Positive (%)	Risky (%)	Protective (%)
	n = 26	n = 587	n = 1	n = 612	n = 55	n = 558
Age group (years)						
12–14	7 (3.4)	197 (96.6)	0	204 (100.0)	14 (6.9)	190 (93.1)
15–17	19 (5.3)	340 (94.7)	0	359 (100.0)	25 (7.0)	334 (93.0)
> 17	0	50 (100)	1 (2.0)	49 (98.0)	16 (32.0)	34 (68.0)
	F = 3.521 ρ = 0.172		F = 11.278 ρ = **0.003**		χ^2^ = 35.348 ρ = ** < 0.001**	
Gender						
Female	7 (2.2)	305 (97.8)	0	312 (100)	10 (3.2)	302 (96.8)
Male	19 (6.3)	282 (93.7)	1 (0.3)	300 (99.7)	45 (15.0)	256 (85.0)
	χ^2^ = 6.244 ρ = **0.012**		F = 1.038 ρ = 0.308		χ^2^ = 23.102 ρ = ** < 0.001**	
School type						
Public	23 (5.3)	411 (94.7)	1 (0.2)	433 (99.8)	35 (8.1)	399 (91.1)
Private	0	179 (100.0)	0	179 (100.0)	20 (11.2)	159 (88.8)
	F = 9.693 ρ = **0.001**		F = 0.413 ρ = 0.520		χ^2^ = 1.500 ρ = 0.221	
Class						
SS 1	10 (4.8)	200 (95.2)	0	211 (100.0)	10 (4.7)	201 (95.3)
SS 2	13 (1.6)	195 (98.4)	0	210 (100.0)	29 (13.8)	181 (86.2)
SS 3	3 (4.2)	187 (95.8)	1 (0.5)	191 (99.5)	16 (8.3)	176 (91.7)
	F = 5.477 ρ = 0.065		F = 2.196 ρ = 0.334		^2^ = 10.741 **ρ = 0.004**	
Subject combination						
Science	6 (2.6)	223 (97.4)	0	225 (100.0)	19 (8.4)	206 (91.6)
Art	13 (5.9)	209 (94.1)	1 (0.4)	229 (99.6)	22 (9.6)	208 (90.4)
Commercial	7 (4.3)	155 (95.7)	0	158 (100.0)	14 (8.9)	144 (91.1)
	χ^2^ = 2.515 ρ = 0.284		F = 2.86 ρ = 0.239		χ^2^ = 0.178 ρ = 0.915	
Marital status						
Single	25 (4.1)	582 (95.9)	1 (0.2)	606 (99.8)	52 (8.6)	555 (91.4)
Married	0	2 (100.0)	0	2 (100.0)	1 (50.0)	1 (50.0)
Others	1 (25.0)	3 (75.0)	0	4 (100.0)	1 (25.0)	3 (75.0)
	F = 4.355 ρ = 0.113		F = 0.01 ρ = 0.995		χ^2^ = 5.574 ρ = 0.062	
Tribe						
Yoruba	22 (4.2)	500 (95.8)	1 (0.2)	521 (99.8)	41 (7.9)	481 (92.1)
Hausa	0	30 (100.0)	0	30 (100.0)	4 (13.3)	26 (86.7)
Igbo	0	15 (100.0)	0	15 (100.0)	2 (13.3)	13 (86.7)
Others	4 (8.7)	42 (91.3)	0	46 (100.0)	8 (17.4)	38 (82.6)
	F = 4.241 ρ = 0.237		F = 0.175 ρ = 0.982		χ^2^ = 5.839 ρ = 0.119	
Religion						
Christianity	10 (5.1)	186 (94.9)	0	196 (100.0)	16 (8.2)	180 (91.8)
Islam	16 (3.8)	401 (96.2)	1 (0.2)	416 (99.8)	39 (9.4)	378 (90.6)
	χ^2^ = 0.525 ρ = 0.469		F = 0.471 ρ = 0.493		χ^2^ = 0.124 ρ = 0.725	
Type of family						
Polygamous	6 (2.9)	201 (97.1)	1 (0.5)	206 (99.5)	15 (7.2)	192 (92.8)
Monogamous	20 (4.9)	386 (95.1)	0	406 (100.0)	40 (9.9)	366 (90.1)
	χ^2^ = 1.388 ρ = 0.239		F = 1.965 ρ = 0.1609		χ^2^ = 1.14 ρ = 0.285	
Parents marital status						
Married	23 (4.2)	523 (95.8)	1 (0.2)	545 (99.8)	47 (8.6)	499 (91.4)
Separated	2 (5.1)	37 (94.9)	0	39 (94.9)	5 (12.8)	34 (87.2)
Widowed	1 (5.3)	18 (94.7)	0	19 (94.7)	2 (10.5)	17 (89.5)
Divorced	0	9 (100.0)	0	9 (100.0)	1 (11.1)	8 (88.9)
	F = 0.524 ρ = 0.913		F = 0.123 ρ = 0.989		F = 0.902 ρ = 0.825	
Father’s employment status						
Unemployed	0	28 (100.0)	0	28 (100.0)	9 (32.1)	19 (67.9)
Self-employed	20 (5.7)	330 (94.3)	0	350 (100.0)	37 (10.6)	313 (89.4)
Civil servant	6 (2.6)	229 (97.4)	1	234 (0.4)	9 (3.8)	226 (96.2)
	F = 4.759 ρ = 0.092		F = 1.611 ρ = 0.446		χ^2^ = 27.111	ρ** < 0.001**
Mother’s employment status						
Unemployed	1 (4.8)	41 (95.2)	1 (2.4)	41 (97.6)	3 (7.1)	39 (92.9)
Self-employed	18 (5.0)	341 (95.0)	0	359 (100.0)	32 (8.9)	327 (91.1)
Civil servant	6 (2.8)	206 (97.2)	0	212 (100.0)	20 (9.4)	192 (90.6)
	F = 1.988 ρ = 0.370		F = 1.895 ρ = 0.387		F = 0.229 ρ = 0.891	
Number of siblings						
0	0	4 (100)	0	4	0	4 (100.0)
1–3	10 (4.0)	237 (96.0)	1	246	19 (7.7)	228 (91.3)
4–6	12 (3.8)	300 (96.2)	0	312	28 (9.0)	284 (91.0)
≥ 7	4 (8.0)	46 (92.0)	0	50	5 (10.0)	45 (90.0)
	F = 2.059 ρ = 0.560		F = 1.484 ρ = 0.685		F = 0.815 ρ = 0.845	
Who respondent lives with						
Parents	22 (4.0)	529 (96.0)	1 (0.2)	550 (99.8)	44 (8.0)	507 (92.0)
Other relatives	4 (7.7)	48 (92.3)	0	52 (100.0)	9 (17.3)	43 (82.7)
Alone	0	6 (100.0)	0	6 (100.0)	1 (16.7)	5 (83.3)
Others	0	4 (100.0)	0	4 (100.0)	1 (25.0)	3 (75.0)
	F = 2.051 ρ = 0.562		F = 0.113 ρ = 0.990		F = 6.774 ρ = 0.079	
Sex education at home						
Yes	20 (4.4)	438 (95.6)	1 (0.2)	457 (99.8)	44 (9.6)	414 (90.4)
	χ^2^ = 0.07 ρ = 0.791		F = 0.339 ρ = 0.560		χ^2^ = 0.893 ρ = 0.344	
Sex education in school						
Yes	23 (4.4)	496 (95.6)	1 (0.2)	518 (99.8)	42 (8.1)	477 (91.9)
	F = 0.301 ρ = 0.583		F = 0.181 ρ = 0.671		χ^2^ = 3.208 ρ = 0.073	

χ^2^—Pearson’s Chi square test, F—Fisher’s exact test, level of significance—p value < 0.05 (bold)

The multivariate analysis showed that females had higher odds of having good SRH knowledge compared with females (AOR = 2.5, 95% CI 1.04, 6.13). Male respondents had less odds of practising protective sexual behaviour (AOR = 0.3, 95% CI 0.15, 0.55). Based on class, respondents in SS2 (AOR = 5.2, 95% CI 1.75, 15.33) and SS3 (AOR = 6.2, 95% CI 1.93, 20.06) had higher odds of practising protective sexual behaviour compared to those in SS1. Respondents whose fathers were self-employed had higher odds (AOR = 3.0, 95% CI 1.12, 8.01) of practising protective sexual behaviour.

### Attrition

At T_1_ and T_2_ the attrition rate in the control group was 3% and 5% respectively, whereas in the intervention group it was 2.5% and 4.2% respectively. Total number of respondents at T_2_ was 1221 (attrition rate of 4.6%).

## Discussion

To the best of our knowledge, this is the first cRCT to assess the effect of an mHealth-based CSE on the SRH knowledge, attitude, and practice of RSB among in-school adolescents in Ilorin, Nigeria. The study was conducted as a proof of concept to promote the national uptake of the FLHE curriculum using mHealth. At baseline, the respondents in the two study groups had comparable sociodemographic characteristics and on average, their baseline SRH knowledge, attitude and RSB profiles were not significantly dissimilar. This suggests that the randomization achieved equivalence in both study groups.

More than half of the respondents in both groups were in the middle adolescence stage (15 to 17 years). Similar studies have shown that most in-school adolescents in senior secondary schools in Nigeria were in the middle/late adolescence stage, and they were unmarried [29, 30]. This stage of adolescence is typified by advanced development of secondary sexual characteristics [31]. During this period, they crave identification to affirm self-image, pre-occupied with fantasies and idealism, and in terms of sexuality, they are testing their ability to attract the opposite sex [31].

At baseline, more than three-fifths and one-third of respondents in the study groups had fair and good SRH knowledge respectively. The survey showed that some adolescents had misconceptions regarding the reproductive system and sexual maturity. Similar studies have also shown that despite having an overall good knowledge of reproductive health, misconceptions regarding the need to have sex multiple times before a girl can get pregnant persist [29, 32, 33]. These misconception could put adolescents at risk of unwanted pregnancies and STIs. Generally, knowledge of STIs, including HIV/AIDS was good in the current study. However, less than half of them were aware of hepatitis B, chlamydia, and genital herpes as STIs. Previous studies have also found knowledge of HIV to be consistently higher than other STIs among adolescents in sub Saharan Africa [34, 35]. HIV/AIDS receives relatively higher attention which may be due to its perceived risk compared to other STIs in Nigeria. Numerous programmes focus on HIV/AIDS among adolescents, including a National HIV Strategy for Adolescents and Young People [36]. This may suggest that adolescents may be less concerned about STIs other than HIV/AIDS which can equally put their reproductive health at risk.

Good knowledge can lead to positive attitude, which can, in turn, lead to less RSB practice. The health belief model hinges on this relationship [37]. About three-quarters of the respondents had positive attitude towards SRH and majority of the students expressed conservative attitudes towards premarital sex. However, the notions of those who had negative attitude should be addressed. Almost a third of the respondents in this study had the perception that having multiple sexual partners is a norm. Only about two thirds of respondents in this study thought contraceptives were important in preventing STIs and another one third of them did not see the need for them or their partners to use a condom. Studies in Nigeria, Ghana and Uganda have shown that a significant proportion of adolescents allude to this perception [38–40]. These findings suggest that a significant number of adolescents have negative SRH attitude which may be detrimental to their reproductive health.

This study found that about one-tenth were sexually active. Of these, the prevalence of risky sexual behaviour (i.e. reported multiple sexual partners, exchange of material gift/money for sex, inconsistent/incorrect/non-use use of condoms at least once, infection by an STI, and sexual debut before the age of 18 years) was found to be more than four-fifths in both study groups. Findings from northern Nigeria and Cape Coast Metropolis Ghana showed that 10% and 13.8% respectively of in-school adolescents were sexually active [29, 41]. However, many studies have reported a significantly higher proportion in other parts of the country and Africa, ranging from 24.7% to 73.8% [34, 42–46]. Scientific evidence has shown a high and increasing rate of sexual activity among adolescents in Nigeria, and an early sexual debut is becoming a concern, particularly among females [42, 47, 48]. Early sexual debut among females has been associated with a high rate of STIs including HIV/AIDS and unintended pregnancies—the latter of which could in turn lead to unsafe abortions, high maternal mortality and infant mortality [4]. Intra-country and inter countries disparities are not unexpected, as these could be linked to rapid urbanization, sociocultural and socioeconomic factors [43, 46]. The higher rates of sexual activity among adolescents in other settings may be linked to differences in data collection methods, relatively higher rates of rapid urbanisation and the cultural differences in these cities compared to Ilorin.

### Intervention effect

The advancement in information technology can be leveraged to improve SRH knowledge. In the current study, the level of completion of the mHealth-based CSE curriculum was high. Within 12 weeks, more than two-thirds of the respondents had completed the course. Within 24 weeks, more than four-fifths had completed the course. The high level of uptake of the curriculum suggests the feasibility of using mHealth-based interventions for SRH interventions among adolescents.

Post intervention (T_1_ and T_2_), there was no statistically significant difference in knowledge, attitude, and sexual behaviour of respondents in the control group. In the intervention group, however, there was a statistically significant increase in the proportion of respondents who had good knowledge of SRH and an increase in mean knowledge score from baseline to T_1_ and T_2_ among respondents in the intervention group. Also, there was a statistically significant increase in the proportion of respondents who had positive attitude at T_1_ and T_2_ and an increase in mean attitude score in the intervention group. However, there was no statistically significant difference in proportion of respondents who practised RSB among respondents in the intervention group at T_1_ and T_2_, and in the mean risky sexual behaviour score compared to baseline in the intervention group.

As put forward by many authors, this finding highlights the importance of CSE in improving adolescents' knowledge and attitude towards SRH [30, 49–51]. In 2007, an internet-based and mobile helpline sexual health information platform was implemented in Nigeria [52]. In 2012, when the programme was evaluated, it was found to be 10–20% more effective as a teaching method than classroom-based teaching of CSE. These findings suggest that mHealth-based interventions are effective in improving the knowledge and attitude of adolescents. Given the current global reality, as seen during the COVID-19 pandemic, online learning plays and will continue to play a significant role in educational institutions. Educational and health institutions in Nigeria should consider implementing mHealth-based strategies in reaching adolescents.

There was no statistically significant decrease in the prevalence of RSB in the control and intervention groups. In contrast, a quasi-experimental study in which in-school adolescents in Ilorin were exposed to a sex education programme found that post-intervention (immediately after the 8-week programme), those in the intervention group reported less at-risk sexual behaviours compared with the control group [49]. The disparity in findings might be due to the difference in study designs and the sample size. The current study was a cRCT with 1280 respondents while the other study was a quasi-experimental study with 24 participants. Furthermore, the findings from this study regarding the effect of the study intervention on RSB is not unexpected due to the interval between implementing the intervention and evaluating behavioural change. Behavioural change among adolescents is not straightforward; it is a spiral process that usually requires ample time and motivation before adopting healthy sexual behaviours [25]. However, using the construct of the health belief model, good knowledge and positive attitude are steps in the right direction towards reducing the practise of risky sexual behaviour [53].

This study showed that being a female was a positive predictor of good SRH knowledge. This is consistent with findings from Iran, where females were found to have better knowledge of SRH compared to their male colleagues [54]. However, this finding is in contrast to the report from Nicaragua, Central America, where adolescent males were more likely to have a better knowledge of SRH because they are more exposed to the media and education [55]. A review of the gender differences in academic performance in the global north and global south found that girls predominantly outperform boys across these settings [56]. In addition, there has been a significant increase in girls' enrolment into schools in Nigeria [57]. These reasons may account for the reported differences.

Being male was found to be a positive predictor of RSB, while being in a more senior class and having a self-employed father were negative predictors. Similar studies have shown that males are more likely to practise RSB compared to females [58, 59]. This may be related to the notion that boys are more adventurous and more likely to take risks than girls [60]. Respondents in more senior classes are more likely to be aware of the consequences of RSB from lessons taught in class which might explain the practice of less RSB among this group compared to those in junior classes. A study conducted in Cameroon corroborates the fact that adolescents whose fathers are unemployed are more likely to practice RSB [61]. Transactional sex has been identified as a means of survival for adolescents from low socioeconomic backgrounds, particularly among females [40, 62]. Adolescents whose fathers are unemployed are likely to have financial constraints and may practice RSB for financial gains.

## Implications for policy and practice

Stakeholders in the Federal and State Ministries of Education are urged to implement an mHealth-based FLHE curriculum in the country. This mode of delivery has the potential to scale-up the country-wide coverage of the curriculum which is currently low due to the associated challenges with the current classroom-based mode of delivery. However, equity considerations should be made in the implementation of this approach. Provision should be made to students without the required technology to ensure equitable access to the curriculum.

Programme managers in governmental and non-governmental organisations are advised to be intentional in targeting adolescent males during the planning and implementation of SRH programmes. Males were found to be more likely to have poor SRH knowledge and practise risky sexual behaviour compared to females. Targeted programmes could help improve the SRH knowledge of males, and also reduce their practice of risky sexual behaviour.

Policymakers and implementers in the educational sector are advised to implement age-appropriate comprehensive sexuality education early in secondary schools. This could address the poor attitude towards SRH found among respondents in lower senior secondary school classes. These stakeholders are also urged to consider the socioeconomic factors of adolescents and their families. The determinants of sexual behaviour are multi-causal, and they include factors beyond the adolescents. This could help address the higher prevalence of risky sexual behaviour among adolescents with unemployed fathers.

## Study limitations

The self-reported nature and sensitivity of the questions asked could have led to respondents under-reporting their sexual behaviours. This was minimised by continuously reassuring the respondents of the confidentiality of their responses and persuading them to be as sincere as possible. Furthermore, during the implementation of the study, students in the control group were expected to continue receiving comprehensive sexuality education as part of the existing curriculum. However, schools were shut down due to the COVID-19 pandemic and this disrupted the regular educational routine of students. This might have had an effect on their performance in the post-intervention evaluation. Post-intervention data from the control and intervention groups were analysed separately to reduce the effect of this limitation. The post intervention effect was measured immediately after the intervention and 3 months after the intervention. Usually, 3 months follow-up period is not long enough to confidently report a sustained behavioural impact of the intervention. Due to the nature of the study, only students who had access to the internet participated in the study. Therefore, findings may not be representative of students without internet access and out-of-school adolescents.

Despite these limitations, however, the study provides useful information for policymakers and stakeholders involved in adolescent SRH in Nigeria. Future studies could consider (1) a study which exposes in-school adolescents to mHealth-based CSE over a longer period of time, to assess the long-term effects of this intervention e.g. 6 months or 12 months (2) a study that involves out-of-school adolescents.

## Conclusion

This study has contributed to the body of knowledge on the effect of mHealth-based CSE among in-school adolescents. A structured mHealth-based intervention delivered over a period of 12 weeks was found to have improved the SRH knowledge and increased positive attitude towards SRH among in-school adolescents who took the course. Such an intervention could help bridge the SRH knowledge and attitude gap among in-school adolescents. Our study findings also suggest that in large scale programmes, males should also be targeted in the implementation of SRH interventions for adolescents. They are less likely to have good SRH knowledge and more likely to practice RSB. Age-appropriate sexuality education curriculum should be implemented as early as possible so that younger adolescents in junior classes can benefit from SRH knowledge which will help them practice protective sexual behaviour. Also, the association between the practice of RSB and unemployment of their fathers, shows the effect of multi-causal factors including socioeconomic factors on the sexual behaviour of adolescents. This study suggests that an improved standard of living in the society especially among parents of adolescents could help reduce risky sexual behaviour among in-school adolescents.

### Supplementary Information

**Additional file 1.** Distribution of selected schools in the control and intervention groups.

**Additional file 2.** Topics covered in the intervention group.

## Data Availability

The datasets used and/or analysed during the current study are available from the corresponding author on reasonable request.

## Abbreviations

AIDS: Acquired immunodeficiency syndrome
RSB: Risky sexual behaviour
CI: Confidence interval
COVID-19: Coronavirus disease 2019
cRCT: Cluster Randomized Controlled Trial
CSE: Comprehensive sexuality education
FLHE: Family Life and HIV Education
HIV: Human immunodeficiency virus
SRH: Sexual and reproductive health
STI: Sexually transmitted infections
T_0_: Assessment at baseline
T_1_: Assessment immediately after the 12-week intervention
T_2_: Assessment 3 months after the intervention
