# Supplementary material for: The effectiveness of an m-Health intervention on the sexual and reproductive health of in-school adolescents: a cluster randomized controlled trial in Nigeria
Source: Reprod Health. 2024 Jan 13;21:6. doi: 10.1186/s12978-023-01735-4 (PMC10788027; doi:10.1186/s12978-023-01735-4)
Supplement: Supplementary file 1 — Additional file 1. Distribution of selected schools in the control and intervention groups. [file 12978_2023_1735_MOESM1_ESM.docx]

Additional File 1: Distribution of Selected Schools in the Control and intervention Groups

| **Allocations** | | **Type of School** | **Total Population of Senior Secondary School Students** | **Number of Respondents Selected for the Study** |
| --- | --- | --- | --- | --- |
| **Control Group** | |  |  |  |
|  | School 1 | Public | 845 | 324 |
|  | School 2 | Public | 407 | 156 |
|  | School 3 | Private | 167 | 64 |
|  | School 4 | Private | 250 | 96 |
| Total | | | 1669 | 640 |
| **Intervention group** | |  |  |  |
|  | School 5 | Public | 834 | 256 |
|  | School 6 | Public | 656 | 203 |
|  | School 7 | Private | 75 | 23 |
|  | School 8 | Private | 515 | 158 |
| Total | | | 2080 | 640 |
