# Supplementary material for: The effectiveness of an m-Health intervention on the sexual and reproductive health of in-school adolescents: a cluster randomized controlled trial in Nigeria
Source: Reprod Health. 2024 Jan 13;21:6. doi: 10.1186/s12978-023-01735-4 (PMC10788027; doi:10.1186/s12978-023-01735-4)
Supplement: Supplementary file 2 — Additional file 2. Topics covered in the intervention group. [file 12978_2023_1735_MOESM2_ESM.docx]

Additional File 2: Topics covered in the intervention group

| **Module** | **Topic** |
| --- | --- |
| Module 1 | Introduction to FHLE |
| Module 2 | Body image |
| Module 3 | Self-esteem |
| Module 4 | Values |
| Module 5 | Puberty and adolescence |
| Module 6 | Reproductive systems |
| Module 7 | Human reproduction |
| Module 8 | Sexually transmitted infections including HIV/AIDS |
| Module 9 | Sexual violence |
| Module 10 | Gender and gender roles |
| Module 11 | Friendship and human emotion (love) |
| Module 12 | Making decisions and setting goals |
